# [2,7-Dimethoxy-8-(4-methylbenzoyl)-1-naphthyl](4-methylphenyl)methanone

**DOI:** 10.1107/S1600536810039620

**Published:** 2010-10-09

**Authors:** Toyokazu Muto, Yuichi Kato, Atsushi Nagasawa, Akiko Okamoto, Noriyuki Yonezawa

**Affiliations:** a Department of Organic and Polymer Materials Chemistry, Tokyo University of Agriculture & Technology, 2-24-16 Naka-machi, Koganei, Tokyo 184-8588, Japan

## Abstract

In the title compound, C_28_H_24_O_4_, the two 4-methyl­benzoyl groups at the 1- and 8-positions of the naphthalene ring system are aligned almost anti­parallel, the dihedral angle between the two phenyl rings being 9.64 (7)°. The dihedral angles between the two phenyl rings and the naphthalene ring system are 71.82 (6) and 71.58 (6)°. In the crystal, inter­molecular C—H⋯O inter­actions between the carbonyl oxygen and aromatic hydrogen are observed.

## Related literature

For the formation reaction of aroylated naphthalene compounds *via* electrophilic aromatic aroylation of 2,7-dimeth­oxy­naphth­alene, see: Okamoto & Yonezawa (2009 ▶). For related structures, see: Nakaema *et al.* (2007 ▶, 2008 ▶); Watanabe *et al.* (2010*a* ▶,*b* ▶).
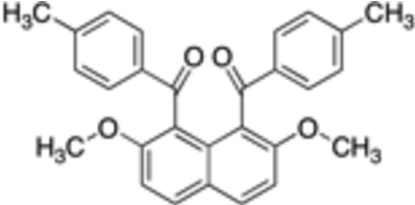


## Experimental

### 

#### Crystal data

C_28_H_24_O_4_
*M* *_r_* = 424.47
Orthorhombic, 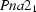

*a* = 20.0334 (3) Å
*b* = 13.4311 (2) Å
*c* = 7.94771 (10) Å
*V* = 2138.49 (5) Å^3^
*Z* = 4
Cu *K*α radiation
μ = 0.70 mm^−1^
*T* = 193 K
0.60 × 0.40 × 0.20 mm

#### Data collection

Rigaku R-AXIS RAPID diffractometer
Absorption correction: numerical (*NUMABS*; Higashi, 1999 ▶) *T*_min_ = 0.604, *T*_max_ = 0.873
33150 measured reflections
2110 independent reflections
2041 reflections with *I* > 2σ(*I*)
*R*_int_ = 0.034

#### Refinement

*R*[*F* ^2^ > 2σ(*F* ^2^)] = 0.034
*wR*(*F* ^2^) = 0.094
*S* = 1.17
2110 reflections
293 parameters
1 restraint
H-atom parameters constrained
Δρ_max_ = 0.18 e Å^−3^
Δρ_min_ = −0.17 e Å^−3^

### 

Data collection: *PROCESS-AUTO* (Rigaku, 1998 ▶); cell refinement: *PROCESS-AUTO*; data reduction: *CrystalStructure* (Rigaku/MSC, 2004 ▶); program(s) used to solve structure: *SIR2004* (Burla *et al.*, 2005 ▶); program(s) used to refine structure: *SHELXL97* (Sheldrick, 2008 ▶); molecular graphics: *ORTEPIII* (Burnett & Johnson, 1996 ▶); software used to prepare material for publication: *SHELXL97*.

## Supplementary Material

Crystal structure: contains datablocks I, global. DOI: 10.1107/S1600536810039620/om2367sup1.cif

Structure factors: contains datablocks I. DOI: 10.1107/S1600536810039620/om2367Isup2.hkl

Additional supplementary materials:  crystallographic information; 3D view; checkCIF report

## Figures and Tables

**Table 1:** Hydrogen-bond geometry (Å, °)

*D*—H⋯*A*	*D*—H	H⋯*A*	*D*⋯*A*	*D*—H⋯*A*
C14—H14⋯O1^i^	0.95	2.52	3.465 (3)	175
C21—H21⋯O2^ii^	0.95	2.38	3.295 (3)	162

Symmetry codes: (i) 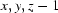; (ii) 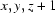.

**Fig. 1.**
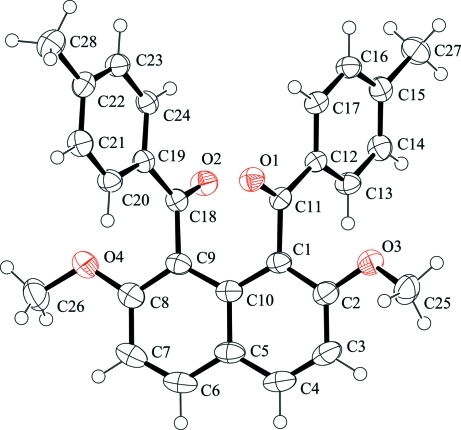
: Molecular structure with the atom-labeling scheme and displacement ellipsoids drawn at the 50% probability level.

**Fig. 2.**
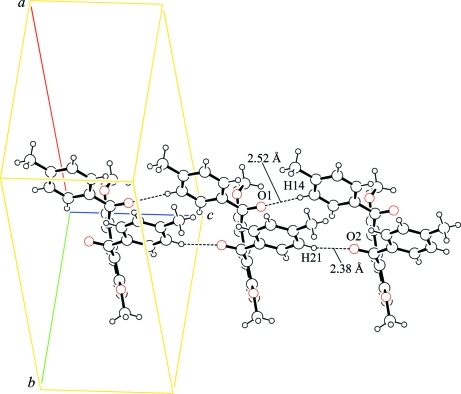
: C—H···O interactions (dashed lines).

## supplementary crystallographic information

### Comment

In the course of our study on selective electrophilic aromatic aroylation of the naphthalene core, *peri*-aroylnaphthalene compounds have proved to be formed regioselectively by the aid of a suitable acidic mediator (Okamoto & Yonezawa, 2009). Recently, we reported the X-ray crystal structures of 1,8-diaroylated 2,7-dimethoxynaphthalene derivatives such as 1,8-bis(4-chlorobenzoyl)-2,7-dimethoxynaphthalene (Nakaema *et al.*, 2007), 1,8-dibenzoyl-2,7-dimethoxynaphthalene (Nakaema *et al.*, 2008), bis(4-bromophenyl)(2,7-dimethoxynaphthalene-1,8-diyl)dimethanone [1,8-bis(4-bromobenzoyl)-2,7-dimethoxynaphthalene] (Watanabe *et al.*, 2010*a*) and (2,7-dimethoxynaphthalene-1,8-diyl)bis(4-fluorophenyl) dimethanone [1,8-bis(4-fluorobenzoyl)-2,7-dimethoxynaphthalene] (Watanabe *et al.*, 2010*b*). The aroyl groups at 1,8-positions of the naphthalene rings in these compounds are oriented in opposite direction. The aromatic rings in the molecule are non-coplanar, resulting in partial disruption of π-conjugation of ring systems. As a part of our continuing studies on the molecular structures of this kind of homologous molecules, the X-ray crystal structure of title compound, *peri*-aroylnaphthalene bearing methyl groups, is discussed in this article.

The molecular structure of the title compound is displayed in Fig 1. Two 4-methylbenzoyl groups are situated in *anti* orientation and are twisted away from the attached naphthalene ring. The interplanar angle between the best planes of the two phenyl rings is 9.64 (7)°. On the other hand, the two interplanar angles between the best planes of the 4-methylphenyl rings and the naphthalene ring are 71.82 (6) and 71.58 (6)°, respectively. The torsion angles between the carbonyl groups and the naphthalene ring [C10—C1—C11—O1 = 68.1 (2)° and C10—C9—C18—O2 = 67.6 (2)°] are larger than those between the carbonyl groups and 4-methylphenyl groups [O1—C11—C12—C13 = -179.18 (15)° and O2—C18—C19—C20 = 176.67 (15)°]. In the molecular packing, the C—H···O hydrogen interactions between the oxygen atoms of the carbonyl groups and the hydrogen atoms of the phenyl rings are observed along the *c* axis [C14—H14···O1 = 2.52 Å and C21—H21···O2 = 2.38 Å] (Fig. 2).

### Experimental

To a 30 ml flask, 4-methylbenzoic acid (8.00 mmol, 1.08 g) and phosphorus pentoxide–methanesulfonic acid mixture (P_2_O_5_–MsOH; 8.0 ml) were placed and stirred at 333 K. To the solution thus obtained, 2,7-dimethoxynaphthalene (4.00 mmol, 0.752 g) was added. After the reaction mixture was stirred at 333 K for 2 h, it was poured into ice-cold water (10 ml) and the mixture was extracted with CHCl_3_ (10 ml × 3). The combined extracts were washed with 2 *M* aqueous NaOH followed by washing with brine. The organic layer was dried over anhydrous MgSO_4_. The solvent was removed under reduced pressure to give a cake (57% yield). The crude product was purified by recrystallization from CHCl_3_-hexane (isolated yield 35%). Furthermore, the isolated product was crystallized from EtOH to give single-crystals.

Spectral data:^1^H NMR δ (300 MHz, CDCl_3_): 2.37 (6*H*, s), 3.67 (6*H*, s), 7.11 (4*H*, d, *J* = 7.8 Hz), 7.19 (2*H*, d, *J* = 8.7 Hz), 7.57 (4*H*, d, *J* = 7.8 Hz), 7.92 (2*H*, d, *J* = 8.7 Hz). ^13^C NMR δ (300 MHz, CDCl_3_): 21.740, 56.495, 111.34, 121.91, 125.58, 128.70, 129.25, 129.74, 131.79, 136.31, 143.17, 156.12, 196.22. IR (KBr): 1655 (C═O), 1607, 1512 (Ar, naphthalene). m.p. = 531.8–534.9 K. Anal. Calcd for C_28_H_24_O_4_; C, 79.22; H, 5,70. Found C, 78.98; H, 5.78.

### Refinement

All the H-atoms could be located in difference Fourier maps. The C-bound H-atoms were subsequently refined as riding atoms, with C—H = 0.95 (aromatic) and 0.98(methyl) Å, and *U_iso_*(H) = 1.2*U_eq_*. Friedel-pair reflections were merged before final refinement because the absolute structure parameter was -0.04 (17). [Merging Friedel-pair data with the MERG 3 instruction in *SHELX97*].

### Crystal data

**Table:**

C_28_H_24_O_4_	*F*(000) = 896
*M**_r_* = 424.47	*D*_x_ = 1.318 Mg m^−^^3^
Orthorhombic, *P**n**a*2_1_	Cu *K*α radiation, λ = 1.54187 Å
Hall symbol: P 2c -2n	Cell parameters from 31782 reflections
*a* = 20.0334 (3) Å	θ = 3.3–68.2°
*b* = 13.4311 (2) Å	µ = 0.70 mm^−^^1^
*c* = 7.94771 (10) Å	*T* = 193 K
*V* = 2138.49 (5) Å^3^	Block, colorless
*Z* = 4	0.60 × 0.40 × 0.20 mm

### Data collection

**Table:**

Rigaku R-AXIS RAPID diffractometer	2110 independent reflections
Radiation source: fine-focus sealed tube	2041 reflections with *I* > 2σ(*I*)
graphite	*R*_int_ = 0.034
Detector resolution: 10.00 pixels mm^-1^	θ_max_ = 68.2°, θ_min_ = 4.0°
ω scans	*h* = −24→24
Absorption correction: numerical (*NUMABS*; Higashi, 1999)	*k* = −16→16
*T*_min_ = 0.604, *T*_max_ = 0.873	*l* = −9→9
33150 measured reflections	

### Refinement

**Table:**

Refinement on *F*^2^	Secondary atom site location: difference Fourier map
Least-squares matrix: full	Hydrogen site location: inferred from neighbouring sites
*R*[*F*^2^ > 2σ(*F*^2^)] = 0.034	H-atom parameters constrained
*wR*(*F*^2^) = 0.094	*w* = 1/[σ^2^(*F*_o_^2^) + (0.0569*P*)^2^ + 0.3088*P*] where *P* = (*F*_o_^2^ + 2*F*_c_^2^)/3
*S* = 1.17	(Δ/σ)_max_ = 0.002
2110 reflections	Δρ_max_ = 0.18 e Å^−^^3^
293 parameters	Δρ_min_ = −0.17 e Å^−^^3^
1 restraint	Extinction correction: *SHELXL*, Fc^*^=kFc[1+0.001xFc^2^λ^3^/sin(2θ)]^-1/4^
Primary atom site location: structure-invariant direct methods	Extinction coefficient: 0.0066 (5)

### Special details

**Table:**

Geometry. All e.s.d.'s (except the e.s.d. in the dihedral angle between two l.s. planes) are estimated using the full covariance matrix. The cell e.s.d.'s are taken into account individually in the estimation of e.s.d.'s in distances, angles and torsion angles; correlations between e.s.d.'s in cell parameters are only used when they are defined by crystal symmetry. An approximate (isotropic) treatment of cell e.s.d.'s is used for estimating e.s.d.'s involving l.s. planes.
Refinement. Refinement of *F*^2^ against ALL reflections. The weighted *R*-factor *wR* and goodness of fit *S* are based on *F*^2^, conventional *R*-factors *R* are based on *F*, with *F* set to zero for negative *F*^2^. The threshold expression of *F*^2^ > σ(*F*^2^) is used only for calculating *R*-factors(gt) *etc*. and is not relevant to the choice of reflections for refinement. *R*-factors based on *F*^2^ are statistically about twice as large as those based on *F*, and *R*- factors based on ALL data will be even larger.

### Fractional atomic coordinates and isotropic or equivalent isotropic displacement parameters (Å^2^)

**Table:**

	*x*	*y*	*z*	*U*_iso_*/*U*_eq_	
O1	0.14819 (8)	0.11387 (12)	0.5115 (2)	0.0340 (4)	
O2	0.14078 (8)	0.32719 (13)	0.2589 (2)	0.0368 (4)	
O3	0.06372 (8)	−0.05143 (12)	0.2673 (3)	0.0449 (4)	
O4	0.03319 (8)	0.46452 (13)	0.4971 (3)	0.0444 (4)	
C1	0.05479 (10)	0.11760 (16)	0.3328 (3)	0.0300 (5)	
C2	0.02338 (11)	0.02950 (17)	0.2891 (3)	0.0361 (5)	
C3	−0.04660 (12)	0.0239 (2)	0.2705 (4)	0.0426 (6)	
H3	−0.0672	−0.0361	0.2345	0.051*	
C4	−0.08397 (11)	0.1059 (2)	0.3048 (3)	0.0420 (6)	
H4	−0.1311	0.1020	0.2935	0.050*	
C5	−0.05501 (10)	0.19654 (19)	0.3567 (3)	0.0369 (5)	
C6	−0.09500 (11)	0.2789 (2)	0.4003 (4)	0.0436 (6)	
H6	−0.1422	0.2727	0.3944	0.052*	
C7	−0.06805 (12)	0.3673 (2)	0.4509 (4)	0.0428 (6)	
H7	−0.0960	0.4215	0.4816	0.051*	
C8	0.00175 (12)	0.37696 (17)	0.4567 (3)	0.0361 (5)	
C9	0.04339 (10)	0.29876 (17)	0.4152 (3)	0.0303 (5)	
C10	0.01624 (10)	0.20501 (17)	0.3670 (3)	0.0309 (5)	
C11	0.12923 (11)	0.11221 (15)	0.3659 (3)	0.0282 (5)	
C12	0.17684 (11)	0.10505 (15)	0.2237 (3)	0.0274 (5)	
C13	0.15505 (11)	0.10444 (17)	0.0569 (3)	0.0330 (5)	
H13	0.1086	0.1075	0.0330	0.040*	
C14	0.20036 (12)	0.09938 (16)	−0.0735 (3)	0.0343 (5)	
H14	0.1847	0.0991	−0.1863	0.041*	
C15	0.26866 (11)	0.09473 (16)	−0.0420 (3)	0.0332 (5)	
C16	0.29032 (11)	0.09517 (17)	0.1250 (3)	0.0343 (5)	
H16	0.3367	0.0916	0.1486	0.041*	
C17	0.24524 (11)	0.10078 (15)	0.2565 (3)	0.0314 (5)	
H17	0.2609	0.1017	0.3693	0.038*	
C18	0.11740 (11)	0.32131 (15)	0.4002 (3)	0.0286 (5)	
C19	0.15908 (11)	0.33410 (15)	0.5519 (3)	0.0280 (5)	
C20	0.13218 (11)	0.33242 (17)	0.7139 (3)	0.0326 (5)	
H20	0.0855	0.3239	0.7283	0.039*	
C21	0.17245 (12)	0.34296 (17)	0.8529 (3)	0.0356 (5)	
H21	0.1532	0.3424	0.9621	0.043*	
C22	0.24135 (12)	0.35449 (16)	0.8353 (3)	0.0335 (5)	
C23	0.26816 (12)	0.35582 (17)	0.6736 (3)	0.0345 (5)	
H23	0.3149	0.3638	0.6594	0.041*	
C24	0.22803 (11)	0.34573 (16)	0.5340 (3)	0.0301 (5)	
H24	0.2473	0.3467	0.4248	0.036*	
C25	0.03540 (14)	−0.14711 (18)	0.2950 (4)	0.0489 (7)	
H25A	0.0694	−0.1983	0.2755	0.059*	
H25B	−0.0020	−0.1573	0.2173	0.059*	
H25C	0.0193	−0.1516	0.4111	0.059*	
C26	−0.00659 (15)	0.5493 (2)	0.5335 (5)	0.0575 (8)	
H26A	0.0224	0.6057	0.5614	0.069*	
H26B	−0.0358	0.5348	0.6292	0.069*	
H26C	−0.0338	0.5661	0.4350	0.069*	
C27	0.31772 (13)	0.0894 (2)	−0.1857 (4)	0.0450 (6)	
H27A	0.2936	0.0769	−0.2909	0.054*	
H27B	0.3495	0.0353	−0.1654	0.054*	
H27C	0.3419	0.1527	−0.1942	0.054*	
C28	0.28470 (13)	0.3658 (2)	0.9887 (4)	0.0442 (6)	
H28A	0.2668	0.3247	1.0802	0.053*	
H28B	0.2853	0.4357	1.0238	0.053*	
H28C	0.3302	0.3442	0.9622	0.053*	

### Atomic displacement parameters (Å^2^)

**Table:**

	*U*^11^	*U*^22^	*U*^33^	*U*^12^	*U*^13^	*U*^23^
O1	0.0338 (8)	0.0407 (9)	0.0276 (9)	−0.0016 (6)	−0.0026 (7)	0.0006 (7)
O2	0.0351 (9)	0.0514 (10)	0.0238 (9)	−0.0047 (7)	0.0037 (7)	0.0015 (7)
O3	0.0372 (8)	0.0403 (9)	0.0573 (11)	−0.0093 (7)	0.0077 (8)	−0.0081 (9)
O4	0.0402 (9)	0.0398 (9)	0.0532 (12)	0.0093 (7)	0.0005 (9)	−0.0030 (8)
C1	0.0260 (10)	0.0401 (11)	0.0237 (10)	−0.0036 (8)	0.0020 (9)	0.0017 (9)
C2	0.0338 (11)	0.0455 (12)	0.0289 (12)	−0.0074 (9)	0.0028 (10)	−0.0013 (10)
C3	0.0353 (11)	0.0551 (14)	0.0374 (13)	−0.0168 (11)	−0.0020 (11)	−0.0008 (12)
C4	0.0258 (10)	0.0635 (16)	0.0368 (14)	−0.0094 (10)	−0.0037 (10)	0.0080 (12)
C5	0.0262 (10)	0.0545 (13)	0.0300 (12)	−0.0008 (10)	−0.0020 (10)	0.0094 (11)
C6	0.0239 (10)	0.0633 (15)	0.0437 (15)	0.0035 (10)	−0.0006 (10)	0.0122 (12)
C7	0.0313 (11)	0.0558 (14)	0.0414 (14)	0.0125 (10)	0.0054 (11)	0.0095 (12)
C8	0.0361 (11)	0.0434 (12)	0.0286 (12)	0.0050 (10)	0.0008 (10)	0.0041 (10)
C9	0.0263 (10)	0.0402 (11)	0.0244 (11)	0.0029 (8)	−0.0013 (9)	0.0046 (9)
C10	0.0267 (10)	0.0443 (12)	0.0218 (10)	−0.0018 (9)	−0.0003 (9)	0.0057 (9)
C11	0.0302 (11)	0.0267 (9)	0.0275 (12)	−0.0023 (8)	−0.0017 (10)	0.0005 (9)
C12	0.0264 (10)	0.0272 (9)	0.0286 (12)	−0.0021 (7)	−0.0009 (9)	−0.0001 (8)
C13	0.0281 (11)	0.0408 (12)	0.0303 (12)	−0.0005 (9)	−0.0048 (9)	0.0018 (10)
C14	0.0387 (12)	0.0381 (11)	0.0262 (11)	0.0005 (9)	−0.0004 (10)	0.0009 (9)
C15	0.0360 (12)	0.0292 (10)	0.0344 (13)	−0.0012 (9)	0.0046 (10)	0.0012 (9)
C16	0.0267 (11)	0.0370 (11)	0.0393 (13)	−0.0026 (8)	0.0011 (10)	0.0001 (10)
C17	0.0294 (10)	0.0331 (10)	0.0317 (12)	−0.0024 (8)	−0.0034 (9)	0.0007 (10)
C18	0.0304 (10)	0.0279 (9)	0.0276 (12)	0.0009 (8)	0.0023 (9)	0.0013 (8)
C19	0.0298 (10)	0.0264 (10)	0.0277 (11)	0.0009 (8)	0.0005 (9)	0.0010 (8)
C20	0.0291 (10)	0.0398 (12)	0.0289 (12)	−0.0008 (9)	0.0018 (9)	0.0002 (10)
C21	0.0404 (12)	0.0405 (12)	0.0259 (12)	−0.0007 (9)	0.0031 (10)	0.0032 (10)
C22	0.0389 (12)	0.0283 (10)	0.0331 (13)	0.0001 (8)	−0.0061 (11)	0.0012 (9)
C23	0.0300 (11)	0.0341 (11)	0.0393 (14)	−0.0002 (9)	−0.0024 (10)	−0.0005 (10)
C24	0.0310 (10)	0.0312 (10)	0.0280 (11)	0.0014 (8)	0.0044 (10)	−0.0004 (9)
C25	0.0531 (15)	0.0413 (12)	0.0523 (17)	−0.0147 (11)	0.0089 (14)	−0.0081 (12)
C26	0.0583 (16)	0.0509 (15)	0.063 (2)	0.0204 (13)	−0.0051 (16)	−0.0145 (15)
C27	0.0419 (13)	0.0522 (14)	0.0408 (15)	0.0002 (10)	0.0110 (12)	0.0014 (12)
C28	0.0500 (15)	0.0459 (13)	0.0368 (14)	−0.0022 (11)	−0.0114 (12)	0.0028 (12)

### Geometric parameters (Å, °)

**Table:**

O1—C11	1.218 (3)	C15—C16	1.396 (4)
O2—C18	1.219 (3)	C15—C27	1.508 (3)
O3—C2	1.366 (3)	C16—C17	1.384 (3)
O3—C25	1.422 (3)	C16—H16	0.9500
O4—C8	1.372 (3)	C17—H17	0.9500
O4—C26	1.420 (3)	C18—C19	1.477 (3)
C1—C2	1.385 (3)	C19—C20	1.395 (3)
C1—C10	1.431 (3)	C19—C24	1.398 (3)
C1—C11	1.516 (3)	C20—C21	1.375 (3)
C2—C3	1.412 (3)	C20—H20	0.9500
C3—C4	1.359 (4)	C21—C22	1.396 (3)
C3—H3	0.9500	C21—H21	0.9500
C4—C5	1.410 (4)	C22—C23	1.393 (3)
C4—H4	0.9500	C22—C28	1.505 (3)
C5—C6	1.409 (4)	C23—C24	1.377 (3)
C5—C10	1.434 (3)	C23—H23	0.9500
C6—C7	1.365 (4)	C24—H24	0.9500
C6—H6	0.9500	C25—H25A	0.9800
C7—C8	1.405 (3)	C25—H25B	0.9800
C7—H7	0.9500	C25—H25C	0.9800
C8—C9	1.381 (3)	C26—H26A	0.9800
C9—C10	1.424 (3)	C26—H26B	0.9800
C9—C18	1.518 (3)	C26—H26C	0.9800
C11—C12	1.482 (3)	C27—H27A	0.9800
C12—C13	1.396 (3)	C27—H27B	0.9800
C12—C17	1.396 (3)	C27—H27C	0.9800
C13—C14	1.379 (3)	C28—H28A	0.9800
C13—H13	0.9500	C28—H28B	0.9800
C14—C15	1.392 (3)	C28—H28C	0.9800
C14—H14	0.9500		
			
C2—O3—C25	117.63 (18)	C15—C16—H16	119.5
C8—O4—C26	118.5 (2)	C16—C17—C12	120.1 (2)
C2—C1—C10	120.23 (19)	C16—C17—H17	119.9
C2—C1—C11	116.73 (18)	C12—C17—H17	119.9
C10—C1—C11	122.48 (18)	O2—C18—C19	121.84 (19)
O3—C2—C1	116.31 (18)	O2—C18—C9	117.41 (19)
O3—C2—C3	122.2 (2)	C19—C18—C9	120.75 (18)
C1—C2—C3	121.5 (2)	C20—C19—C24	118.5 (2)
C4—C3—C2	118.9 (2)	C20—C19—C18	122.2 (2)
C4—C3—H3	120.6	C24—C19—C18	119.2 (2)
C2—C3—H3	120.6	C21—C20—C19	120.8 (2)
C3—C4—C5	122.1 (2)	C21—C20—H20	119.6
C3—C4—H4	118.9	C19—C20—H20	119.6
C5—C4—H4	118.9	C20—C21—C22	120.7 (2)
C6—C5—C4	121.05 (19)	C20—C21—H21	119.6
C6—C5—C10	119.3 (2)	C22—C21—H21	119.6
C4—C5—C10	119.6 (2)	C23—C22—C21	118.4 (2)
C7—C6—C5	122.03 (19)	C23—C22—C28	121.6 (2)
C7—C6—H6	119.0	C21—C22—C28	120.0 (2)
C5—C6—H6	119.0	C24—C23—C22	121.1 (2)
C6—C7—C8	118.9 (2)	C24—C23—H23	119.4
C6—C7—H7	120.5	C22—C23—H23	119.4
C8—C7—H7	120.5	C23—C24—C19	120.4 (2)
O4—C8—C9	115.50 (19)	C23—C24—H24	119.8
O4—C8—C7	122.9 (2)	C19—C24—H24	119.8
C9—C8—C7	121.5 (2)	O3—C25—H25A	109.5
C8—C9—C10	120.40 (18)	O3—C25—H25B	109.5
C8—C9—C18	117.19 (19)	H25A—C25—H25B	109.5
C10—C9—C18	121.89 (18)	O3—C25—H25C	109.5
C9—C10—C1	124.76 (17)	H25A—C25—H25C	109.5
C9—C10—C5	117.74 (19)	H25B—C25—H25C	109.5
C1—C10—C5	117.5 (2)	O4—C26—H26A	109.5
O1—C11—C12	121.7 (2)	O4—C26—H26B	109.5
O1—C11—C1	118.1 (2)	H26A—C26—H26B	109.5
C12—C11—C1	120.25 (19)	O4—C26—H26C	109.5
C13—C12—C17	118.9 (2)	H26A—C26—H26C	109.5
C13—C12—C11	121.6 (2)	H26B—C26—H26C	109.5
C17—C12—C11	119.5 (2)	C15—C27—H27A	109.5
C14—C13—C12	120.5 (2)	C15—C27—H27B	109.5
C14—C13—H13	119.7	H27A—C27—H27B	109.5
C12—C13—H13	119.7	C15—C27—H27C	109.5
C13—C14—C15	120.9 (2)	H27A—C27—H27C	109.5
C13—C14—H14	119.5	H27B—C27—H27C	109.5
C15—C14—H14	119.5	C22—C28—H28A	109.5
C14—C15—C16	118.4 (2)	C22—C28—H28B	109.5
C14—C15—C27	120.4 (2)	H28A—C28—H28B	109.5
C16—C15—C27	121.2 (2)	C22—C28—H28C	109.5
C17—C16—C15	121.0 (2)	H28A—C28—H28C	109.5
C17—C16—H16	119.5	H28B—C28—H28C	109.5
			
C25—O3—C2—C1	153.3 (3)	C10—C1—C11—O1	67.9 (3)
C25—O3—C2—C3	−25.9 (4)	C2—C1—C11—C12	76.4 (3)
C10—C1—C2—O3	−176.7 (2)	C10—C1—C11—C12	−112.1 (2)
C11—C1—C2—O3	−5.0 (3)	O1—C11—C12—C13	−179.2 (2)
C10—C1—C2—C3	2.5 (4)	C1—C11—C12—C13	0.9 (3)
C11—C1—C2—C3	174.1 (2)	O1—C11—C12—C17	−0.5 (3)
O3—C2—C3—C4	175.7 (3)	C1—C11—C12—C17	179.60 (18)
C1—C2—C3—C4	−3.4 (4)	C17—C12—C13—C14	0.2 (3)
C2—C3—C4—C5	0.7 (4)	C11—C12—C13—C14	178.9 (2)
C3—C4—C5—C6	−176.6 (3)	C12—C13—C14—C15	0.0 (3)
C3—C4—C5—C10	2.8 (4)	C13—C14—C15—C16	0.1 (3)
C4—C5—C6—C7	−180.0 (3)	C13—C14—C15—C27	−179.9 (2)
C10—C5—C6—C7	0.6 (4)	C14—C15—C16—C17	−0.4 (3)
C5—C6—C7—C8	1.1 (4)	C27—C15—C16—C17	179.5 (2)
C26—O4—C8—C9	177.1 (3)	C15—C16—C17—C12	0.7 (3)
C26—O4—C8—C7	−0.4 (4)	C13—C12—C17—C16	−0.6 (3)
C6—C7—C8—O4	176.4 (2)	C11—C12—C17—C16	−179.3 (2)
C6—C7—C8—C9	−1.1 (4)	C8—C9—C18—O2	−104.1 (3)
O4—C8—C9—C10	−178.4 (2)	C10—C9—C18—O2	67.6 (3)
C7—C8—C9—C10	−0.8 (4)	C8—C9—C18—C19	76.7 (3)
O4—C8—C9—C18	−6.6 (3)	C10—C9—C18—C19	−111.6 (2)
C7—C8—C9—C18	171.0 (2)	O2—C18—C19—C20	176.7 (2)
C8—C9—C10—C1	−175.5 (2)	C9—C18—C19—C20	−4.1 (3)
C18—C9—C10—C1	13.1 (4)	O2—C18—C19—C24	−5.0 (3)
C8—C9—C10—C5	2.5 (3)	C9—C18—C19—C24	174.19 (18)
C18—C9—C10—C5	−169.0 (2)	C24—C19—C20—C21	0.6 (3)
C2—C1—C10—C9	179.0 (2)	C18—C19—C20—C21	178.9 (2)
C11—C1—C10—C9	7.9 (4)	C19—C20—C21—C22	−0.7 (3)
C2—C1—C10—C5	1.1 (4)	C20—C21—C22—C23	0.5 (3)
C11—C1—C10—C5	−170.1 (2)	C20—C21—C22—C28	−179.9 (2)
C6—C5—C10—C9	−2.4 (4)	C21—C22—C23—C24	−0.2 (3)
C4—C5—C10—C9	178.2 (2)	C28—C22—C23—C24	−179.8 (2)
C6—C5—C10—C1	175.8 (2)	C22—C23—C24—C19	0.1 (3)
C4—C5—C10—C1	−3.7 (4)	C20—C19—C24—C23	−0.4 (3)
C2—C1—C11—O1	−103.5 (3)	C18—C19—C24—C23	−178.7 (2)

### Hydrogen-bond geometry (Å, °)

**Table:**

*D*—H···*A*	*D*—H	H···*A*	*D*···*A*	*D*—H···*A*
C14—H14···O1^i^	0.95	2.52	3.465 (3)	175
C21—H21···O2^ii^	0.95	2.38	3.295 (3)	162

Symmetry codes: (i) *x*, *y*, *z*−1; (ii) *x*, *y*, *z*+1.
